# Genetic associations with 25-hydroxyvitamin D deficiency in HIV-1-infected youth: fine-mapping for the *GC/DBP* gene that encodes the vitamin D-binding protein

**DOI:** 10.3389/fgene.2013.00234

**Published:** 2013-11-14

**Authors:** Travis R. Porter, Xuelin Li, Charles B. Stephensen, Kathleen Mulligan, Brandy Rutledge, Patricia M. Flynn, Jorge Lujan-Zilbermann, Rohan Hazra, Craig M. Wilson, Peter L. Havens, Jianming Tang, Adolescent MedicineTrials Network for HIV/AIDS Interventions (ATN) 063 study team

**Affiliations:** ^1^Department of Epidemiology, University of Alabama at BirminghamBirmingham, AL, USA; ^2^Department of Medicine, University of Alabama at BirminghamBirmingham, AL, USA; ^3^Western Human Nutrition Research Center, U.S. Department of Agriculture-Agricultural Research ServiceDavis, CA, USA; ^4^Division of Endocrinology, University of California at San FranciscoSan Francisco, CA, USA; ^5^WestatRockville, MD, USA; ^6^Department of Infectious Diseases, St. Jude Children’s Research HospitalMemphis, TN, USA; ^7^Division of Pediatric Infectious Diseases, University of South Florida College of MedicineTampa, FL, USA; ^8^Eunice Kennedy Shriver National Institute of Child Health and Human DevelopmentBethesda, MD, USA; ^9^Children’s Research Institute, Medical College of WisconsinMilwaukee, WI, USA; ^10^Children’s Hospital of Wisconsin, Medical College of WisconsinMilwaukee, WI, USA

**Keywords:** antiretroviral, genetics, HIV-1, race, youth, vitamin D

## Abstract

Serum 25-hydroxyvitamin D [25(OH)D] is often deficient (<12 ng/ml) or insufficient (<20 ng/ml) in youth living with human immunodeficiency virus type 1 infection (YLH). Based on evidence from multiple genome-wide association studies, we hypothesized that genetic factors associated with 25(OH)D deficiency should be readily detectable in YLH even when controlling for other known factors, including use of the antiretroviral drug efavirenz (EFV). Genotyping by bi-directional sequencing targeted 15 single nucleotide polymorphisms (SNPs) at the *GC*/*DBP* locus, with a focus on coding and regulatory variants, as well as those repeatedly reported in the literature. Three intronic SNPs (rs222016, rs222020, and rs222029) in a conserved haplotype block had unequivocal association signals (false discovery rate ≤ 0.006). In particular, the minor allele G for rs222020 was highly unfavorable among 192 YLH (99 African–Americans and 93 others), as gauged by relatively low likelihood for 25(OH)D sufficiency at enrollment (odds ratio = 0.31, *p* = 9.0 × 10^-4^). In a reduced multivariable model, race, season, latitude, body mass index, exposure to EFV, and rs222020-G were independent factors that collectively accounted for 38% of variance in the log_10_-transformed 25(OH)D concentration (*p* < 0.0001). Interaction terms were evident for rs222020-G × season (*p* < 0.001), latitude × season (especially fall and winter; *p* < 0.01), and race × EFV use (*p* = 0.024). Overall, variance in serum 25(OH)D is substantially attributable to multiple factors, but the exact contribution of genetic and non-genetic factors can be obscured by partial overlaps and frequent interactions.

## INTRODUCTION

The vitamin D pathway has a wide range of pathophysiological implications, with documented roles in bone metabolism, renal function, cardiovascular disease, and immune responses (Chocano-Bedoya and Ronnenberg, 2009; Ramagopalan et al., 2009; Razzaque, 2009). In the U.S. general population, suboptimal serum 25-hydroxyvitamin D [25(OH)D] concentration (<20 ng/ml)] is highly prevalent (Looker et al., 2008; Ginde et al., 2009). Factors associated with serum 25(OH)D status include race (skin color), seasonal (environmental) fluctuation, behavior, and genetic pre-disposition (Zwart et al., 2007; Bouillon, 2010; Bu et al., 2010; Karohl et al., 2010; Rosen, 2011; Schlingmann et al., 2011; Vimaleswaran et al., 2013).

Suboptimal serum 25(OH)D is seen in 54% of youth living with human immunodeficiency virus type 1 (HIV) infection (YLH; Havens et al., 2012a, b). The problem with 25(OH)D insufficiency (<20 ng/ml) or deficiency (<12 ng/ml) can be exacerbated by long-term use of antiretroviral drugs, especially efavirenz (EFV) that is known to interfere with 25(OH)D metabolism (Childs et al., 2012; Panayiotopoulos et al., 2013). Longitudinal data from YLH with and without vitamin D supplementation can provide an important platform for dissecting multifactorial influences on the vitamin D pathway, including pre-vitamin D transport mediated by the vitamin D-binding protein (VDBP; Schlingmann et al., 2011).

The *GC*/*DBP* gene^1^ encoding VDBP is mapped to chromosome 4q12-q13, with hundreds of known single nucleotide polymorphisms (SNPs). When 25(OH)D concentration is analyzed as a trait for vitamin D status, both genome-wide association studies (Wang et al., 2010) and candidate gene approaches (Bu et al., 2010) have consistently pointed to the potential importance of *GC* SNP variants. In an attempt to confirm the *GC* genotypes associated with 25(OH)D deficiency, our work here provides further evidence to justify fine-mapping for the *GC* locus in YLH populations.

## MATERIALS AND METHODS

### STUDY POPULATION

YLH (18–25 years old) represented two self-identified racial groups (African–American (AAs) and others) participating in a randomized, double-blind, placebo-controlled, multicenter trial (NCT00490412^2^) within the Adolescent Medicine Trials Network for HIV/AIDS Interventions (ATN; Havens et al., 2012a, b). The research protocols, including procedures for written informed consent, were approved by the Institutional Review Board (IRB) at 16 ATN clinics and 19 International Maternal Pediatric Adolescent AIDS Clinical Trials (IMPAACT) sites in the United States and Puerto Rico. Ancillary studies summarized here were further approved by the IRB at University of Alabama at Birmingham (UAB).

### INTERVENTION AND OUTCOME MEASURES

All participants were treated with ≥3 antiretrovirals (ARVs) for ≥90 days and with plasma HIV-1 RNA (viral load) <5,000 copies/mL within 60 days. After screening, subjects free of renal disease, pregnancy, and medicines that may affect bone mineral density, interfere with vitamin D absorption, or cause renal toxicity were enrolled into two relatively equal groups based on their ARV regimens (with or without tenofovir disoproxil fumarate, TDF). Within each group, eligible participants were randomized to receive vitamin D supplementation or placebo every 4 weeks for three doses. Serum 25(OH)D concentration was measured at baseline (week 0) and at study week 12 as the primary outcomes for analyses here.

### CANDIDATE LOCI AND GENOTYPING

Earlier reports on phenotypes related to vitamin D (Bu et al., 2010; Wang et al., 2010; Levin et al., 2012), including bone mineral density and fracture (Cho et al., 2009; Richards et al., 2009; Rivadeneira et al., 2009), have revealed various loci with modest associations (as judged by effect sizes instead of *p* values). For this study, SNP selection focused on the most promising *GC*/*DBP* locus that encodes vitamin D-binding protein. SNPs reported repeatedly in the literature were considered first, followed by flanking SNPs (to facilitate analysis of linkage disequilibrium, LD) and SNPs found in coding and regulatory sequences. Using DNA extracted from Isohelix buccal swabs (Cell Projects Ltd., Kent, UK), all SNP genotypes were resolved by bi-directional DNA sequencing using the gold-standard Sanger chemistry (Polymorphic DNA Technologies, Inc., Alameda, CA, USA). For SNPs with minor allele frequencies (MAF) exceeding 0.05, the pairwise LD patterns were tested using the HaploView program (Barrett et al., 2005).

### STATISTICAL ANALYSES

The study population was first grouped by race (AAs vs. others) for comparison of baseline (week 0) characteristics, with Wilcoxon test, Student *t*-test, and Chi-squared test applied to appropriate measurements. Subsequent analyses focused on three specific aims. Aim 1 was to demonstrate that serum 25(OH)D concentration is a relatively stable phenotype in YLH. Measurements at baseline and at week 12 were compared in participants in the placebo group (who did not receive vitamin D supplementation), using Spearman method (rho) and Pearson’s correlation coefficient (*r*; before and after log_10_-transformation/“normalization,” respectively). Aim 2 was to identify individual SNP genotypes associated with three clinically relevant 25(OH)D categories at baseline: <12 ng/ml (deficiency), ≥12–<20 ng/ml (insufficiency) and >20 ng/ml (sufficiency), using the ordinal logistic regression models adjusted for non-genetic factors (age, sex, and race). All relationships with statistical significance (*p* < 0.05) and low false discovery rate (FDR; *q* < 0.05) were included in multivariable models. Aim 3 was to quantify multifactorial influences on serum 25(OH)D, when log_10_-transformed serum 25(OH)D was analyzed as a continuous outcome in generalized linear models (GLMs). The summary statistics focused on relative effect sizes (regression beta and *R*^2^ values) attributable to genetic factors (SNP genotypes), demographic features (age, sex, and race), body mass index (BMI), environmental factors (season and latitude), and exposure to EFV. Similar approaches have been applied earlier to analyses of quantitative traits related to HIV infection (Yue et al., 2013). Whenever possible, secondary (exploratory) models were evaluated for AAs and other races separately.

## RESULTS

### CHARACTERISTICS OF STUDY POPULATION BY RACE

A total of 192 YLH subjects had sufficient data for analyses, with relative equal representation of AAs (*n* = 99) and others (*n* = 93; **Table 1**). At baseline (week 0), these groups were similar (*p* > 0.20) in terms of age, female to male sex ratio (0.62 vs. 0.58), latitude of residency, enrollment seasons, randomization to vitamin D supplementation (49.5 vs. 52.3%), exposure to EFV (45.5 vs. 37.6%), and CD4^+^ T-cell (CD4) count (505 ± 149 vs. 550 ± 224 cells/μl of blood). In addition, similar proportions of AAs (13.0%) and others (17.5%) had severe immunodeficiency at baseline (CD4 count < 350 cells/μl). On the other hand, AAs differed from others in serum 25(OH)D concentrations both at baseline (*p* < 0.0001) and at week 12 (*p* < 0.01).

**Table 1:** Main characteristics of the study population, after stratification by race/ethnicity.

Characteristics^a^	AAs (*n* = 99)	Others (*n* = 93)	*P*^e^
**Enrollment season: *n* (%)**			
Winter	23 (23.2)	18 (19.4)	
Spring	30 (30.3)	32 (34.4)	
Summer	24 (24.3)	23 (24.7)	
Fall	22 (22.2)	20 (21.5)	
**At baseline (week 0)**			
Age (year): mean ± SD	20.8 ± 2.0	20.9 ± 2.0	–
BMI (kg/m^2^): mean ± SD	26.3 ± 7.7	24.5 ± 5.5	0.058
Sex ratio (F/M)	0.62 (38/61)	0.58 (34/59)	–
Latitude: *n* (%)			0.067
≤35°	30 (30.3)	41 (44.1)	
>35° and ≤40°	26 (26.3)	14 (15.0)	
>40°	43 (43.4)	38 (40.9)	
25(OH)D (ng/ml): median (IQR)	14.7 (10.0 - 21.0)	23.8 (17.4 - 29.9)	<0.0001^f^
log_10_ 25(OH)D: mean ± SD	1.17 ± 0.23	1.37 ± 0.19	<0.0001
25(OH)D < 20 ng/ml: *n* (%)	71 (71.7)	32 (34.4)	<0.0001
Vitamin D supplementation (50,000 U/month)	49 (49.5)	49 (52.7)	–
Exposure to EFV: *n* (%)	45 (45.5)	35 (37.6)	–
CD4 count (cells/μl): mean ± SD^b^	505 ± 149	550 ± 224	–
CD4 < 350 cells/μl: *n* (%)^b^	7 (13.0)	10 (17.5)	–
**At week 12**			
25(OH)D (ng/ml): median (IQR)	24.3 (15.2 - 30.7)	27.3 (22.2 - 36.3)	0.005^f^
log_10_ 25(OH)D: mean ± SD	1.34 ± 0.26	1.46 ± 0.19	0.0006
25(OH)D < 20 ng/ml: *n* (%)	33 (35.9)	17 (18.7)	0.009
CD4 count (cells/μl): mean ± SD^c^	521 ± 161	617 ± 280	0.051
CD4 < 350 cells/μl: *n* (%)^c^	5 (11.6)	6 (12.2)	–
**Between visits^d^**			
Stability of 25(OH)D: rho (*p*)	0.69 (<0.0001)	0.63 (<0.0001)	–
Stability of log_10_ 25(OH)D: *r* (*p*)	0.73 (<0.0001)	0.77 (<0.0001)	–

a AAs, African–Americans; BMI, body mass index; F, female; M, male; 25(OH)D, serum 25-hydroxyvitamin D concentration; IQR, interquartile range; SD, standard deviation of the mean; EFV, efavirenz; CD4 count, CD4^+^ T-cell count in peripheral blood.

b Partial data for 54 AAs and 57 others at week 0.

c Partial data for 43 AAs and 49 others at week 12.

d Restricted to the subset of subjects (46 AAs and 42 others) randomized to the placebo group. Six subjects (four AAs and two others) are excluded because of missing data at week 12.

e All p values > 0.20 are omitted (–).

e By Wilcoxon test; all other comparisons are done with t-test and Chi-squared test.

### STABILITY OF SERUM 25(OH)D CONCENTRATION OVER A 12-WEEK PERIOD

**FIGURE 1:**
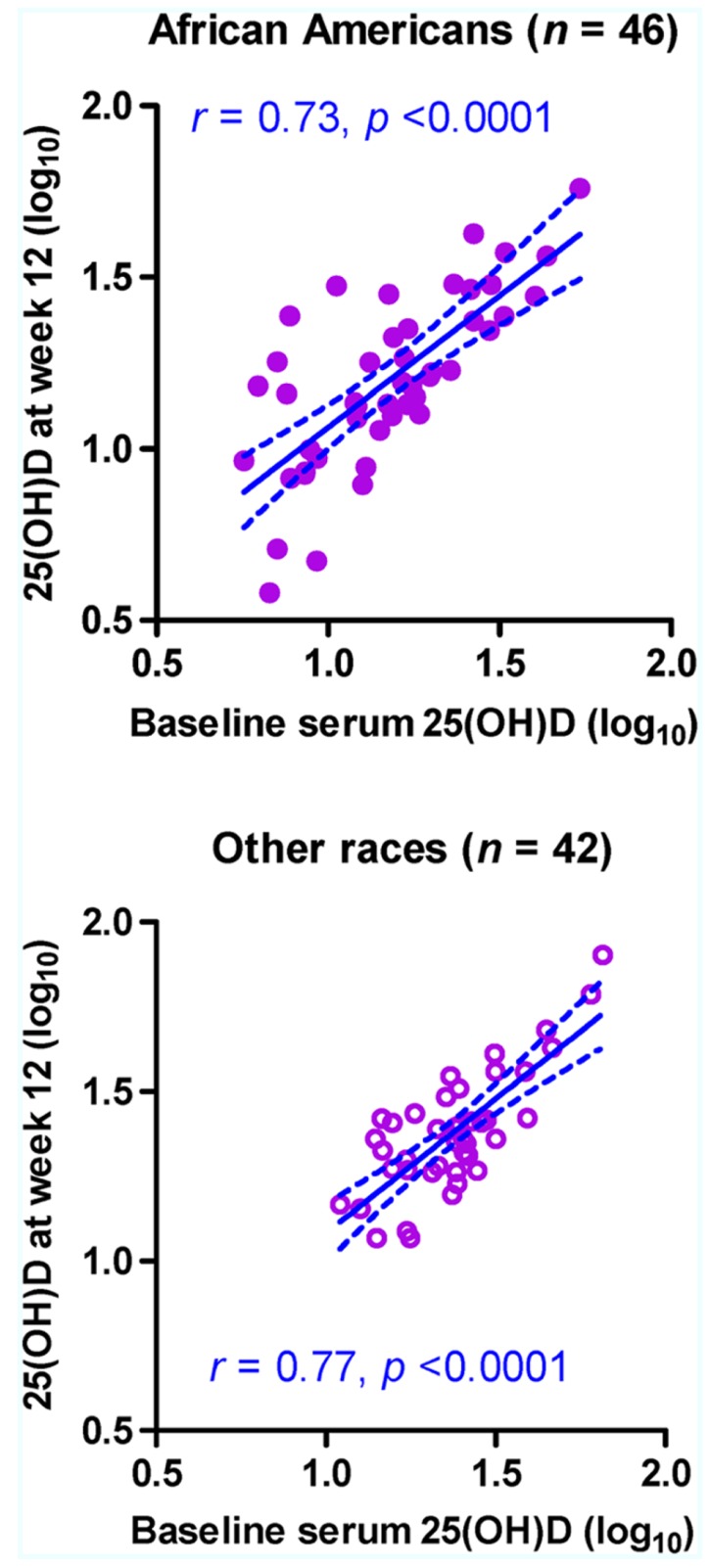
**Stability of serum 25-hydroxyvitamin D [25(OH)D] concentration (ng/ml) in 88 HIV-1-infected youth who did not receive randomized vitamin D supplementation over a 12-week period.** Measurements at baseline (week 0) and at study week 12 are shown for 46 African–Americans (AAs) and 42 non-AA subjects (others). The predicted slope and its 95% confidence intervals in each subgroup are represented by sold and dotted lines, respectively. Six subjects (four AAs and two others) with missing data at week 12 are excluded.

In a subset of subjects (46 AAs and 42 others) who were randomized to the placebo group, log_10_-transformed 25(OH)D concentrations were moderately stable between the two visits regardless of race (**Figure 1**), with Pearson *r* values ranging from 0.73 in AAs (*p* < 0.0001) to 0.77 in others (*p* < 0.0001; *p* > 0.50 between the two *r* values). Statistical adjustments for other factors slightly improved the *r* values. For example, when season was treated as a covariate, the adjusted *r* value became 0.74 in AAs (*p* < 0.0001) and 0.83 in others (*p* < 0.0001; *p* = 0.322 between the two adjusted *r* values). For 25(OH)D concentrations without log_10_-transformation, rank correlation between visits (Spearman rho values) ranged from 0.69 in AAs (*p* < 0.0001) to 0.63 in others (*p* < 0.0001; *p* > 0.50 between the two rho values).

### SCREENING FOR INFORMATIVE *GC* SNPs

**FIGURE 2:**
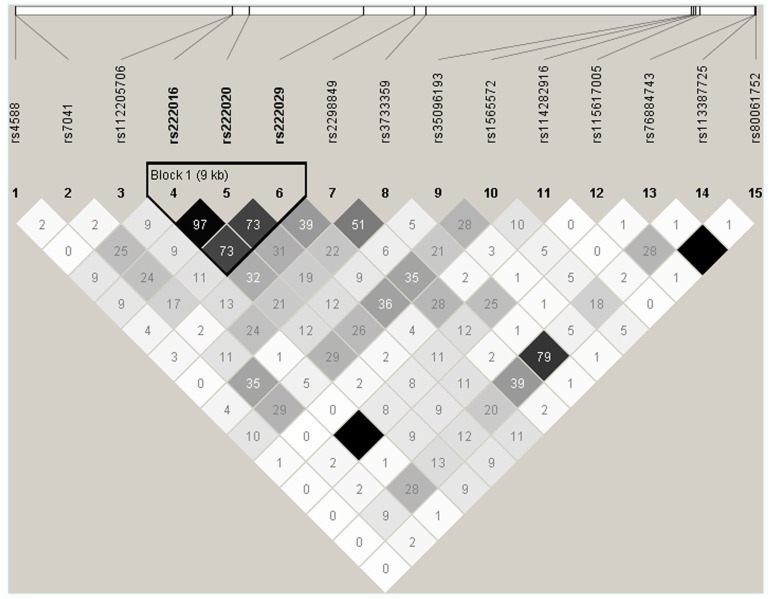
**Patterns of pairwise linkage disequilibrium (LD) among 15 *GC* SNPs used for screening and estimating statistical power.** Each pairwise comparison of SNP genotypes (defined by direct sequencing) is captured by an *r*^2^ value (×100). Similar results are obtained from analyses of SNP genotypes in AAs and other races (excluding SNPs rs112205706 and rs115617005). By default, absolute LD (*r*^2^ = 1.00) is indicated by a dark square.

DNA sequencing based on 91 samples with most DNA (47 AAs and 44 others) identified 15 informative SNPs with MAF ≥ 0.05 in the overall study population (**Table 2**). All but one SNP (rs114282916) showed differential distribution between the two racial groups (AAs and others). Most SNPs had weak pairwise LD in both racial groups, but three intronic SNPs (rs222016, rs222020, and rs222029) were within a conserved haplotype block (**Figure 2**). Additional SNPs dismissed based on rarity of minor alleles (singleton to MAF < 0.05) included rs9016, rs3737553, rs80324156, rs114737000, rs6843222, and 10 polymorphisms not captured in the dbSNP database (last accessed in April 2013).

**Table 2:** Minor allele frequency for 15 *GC* SNPs resolved by DNA sequencing.

SNP ID^a^	Alleles	Location	Minor allele frequency		
			Overall	African–Americans	Others
rs4588^b^	C/A	Exon 11	0.177	0.112	0.247
rs7041^b^	T/G	Exon 11	0.310	0.143	0.489
rs112205706	G/A	Intron	0.053	0.098	0.011
rs222016	A/G	Intron	0.383	0.565^c^	0.205
rs222020^b^	A/G	Intron	0.385	0.535^c^	0.226
rs222029	A/G	Intron	0.325	0.467	0.182
rs2298849	A/G	Intron	0.309	0.391	0.227
rs3733359	G/A	Promoter	0.184	0.255	0.102
rs35096193	G/T	Promoter	0.210	0.087	0.341
rs1565572	G/T	Promoter	0.479	0.282	0.670^c^
rs114282916	C/T	Promoter	0.105	0.117	0.102
rs115617005	T/C	Promoter	0.051	0.107	0
rs76884743	A/T	Promoter	0.053	0.096	0.011
rs113387725	C/T	Promoter	0.163	0.213	0.102
rs80061752	C/T	Promoter	0.053	0.096	0.011

a Sorted by location on chromosome 4q (see **Figure 2**). Six more known SNPs (rs9016, rs3737553, rs80324156, rs114737000, rs6843222, and rs71213589) are dismissed for rarity of their minor alleles.

b These SNPs are sequenced for the entire cohort, while others are dropped after the screening phase (based on patterns of linkage disequilibrium and estimates of statistical power).

c Minor and major alleles are switched between the two racial groups.

In univariable models testing three clinically relevant 25(OH)D levels at baseline: <12 ng/ml (deficiency), ≥12–<20 ng/ml (insufficiency), and >20 ng/ml (sufficiency), seven SNPs showed promising trend (*p* < 0.05 and *q* ≤ 0.10) for associations in dominant models, with proportional odds ratios (pOR) ranging from 0.17 (rs222016 and rs222020, unfavorable) to 3.41 (rs7041, favorable) and *q* values from 0.002 (rs222016 and rs222020) to 0.10 (rs35096193, favorable; **Table 3**). Among the top four SNPs with *q* < 0.05, rs7041 has known associations with vitamin D status and related outcomes (Fang et al., 2009; Wood et al., 2011). Three other SNPs in strong LD (**Figure 2**) could be represented by rs222020, which has been associated with vitamin D status and related outcomes as well (Bu et al., 2010; Xu et al., 2010; Jung et al., 2011; Zhang et al., 2012). Further genotyping in the rest of the study cohort focused on rs7041 (a coding SNP) and rs222020 (an intronic SNP).

**Table 3:** Univariable analyses of seven *GC* SNPs using baseline (week 0) serum 25(OH)D concentration as three ordinal categories (deficiency, insufficiency, and sufficiency).

*GC* SNP genotypes	*n* (AA + other)	pOR^a^	*p*	FDR (*q*)^c^
rs7041 (GG + GT vs. TT)	90 (46 + 44)	3.41	0.003	0.010
rs222016^b^ (GG+AG vs. AA)	90 (46 + 44)	0.17	0.0003	0.002
rs222020^b^ (GG + AG vs. AA)	91 (47 + 44)	0.17	0.0002	0.002
rs222029^b^ (GG + AG vs. AA)	90 (46 + 44)	0.24	0.001	0.006
rs35096193 (TT + TG vs. GG)	90 (46 + 44)	2.35	0.047	0.100
rs76884743 (TT + AT vs. AA)	91 (47 + 44)	0.24	0.025	0.060
rs80061752 (TT + TC vs. CC)	91 (47 + 44)	0.24	0.025	0.060

a Favorable effect (association with vitamin D sufficiency) is reflected by a value of ±1.0 for the proportional odds ratio (pOR).

b These SNPs form a single haplotype block regardless of racial background (see **Figure 2)**.

c False discovery rate (FDR) is based on p values from analyses of all 15 SNPs shown in **Table 2**.

### UNIVARIABLE AND MULTIVARIABLE MODELS FOR TWO GC SNPs (rs222020 and rs7041) IN THE ENTIRE COHORT

Both rs7401-G and rs222020-G were associated with baseline serum 25(OH)D categories in univariable models (pOR = 2.32 and 0.31, *p* = 0.008 and 9.0 × 10^-4^, respectively). After statistical adjustment for demographic features (sex, age, and race) and exposure to EFV, rs7041 allele G was no longer a predictor (adjusted pOR = 1.08 and *p* = 0.827), while rs222020 allele G remained predictive of serum 25(OH)D categories (pOR = 0.45 and *p* = 0.014). However, further adjustments for BMI and environmental factors (latitude and enrollment season) diminished the association of rs222020-G (adjusted *p* = 0.069; **Table 4**). The strong independent predictors included race (*p* < 0.0001), enrollment season (*p* < 0.0001), latitude of residence (*p* < 0.001), BMI (*p* = 0.002), and use of EFV (*p* = 0.006). Summer had the most dramatic impact on seasonal fluctuation in serum 25(OH)D (pOR = 8.23, *p* < 0.0001), while fall and spring were also quite favorable against winter (pOR = 4.90 and 3.55, respectively).

**Table 4:** Independent predictors of serum 25(OH)D concentration as three ordinal categories (deficiency, insufficiency, and sufficiency).

Independent factors	Relative effect^c^ in a joint (multivariable) model		
	pOR	95% CI	*p*
rs222020-G^a^	0.54	0.27 - 1.05	0.069
Being African–American	0.21	0.11 - 0.41	<0.0001
Enrollment season			<0.0001
Spring^b^	3.55	1.53 - 8.20	0.003
Summer^b^	8.23	3.22 - 21.04	<0.0001
Fall^b^	4.90	1.98 - 12.15	<0.0001
Latitude of residence (trend)	0.52	0.63 - 0.74	<0.001
Body mass index	0.93	0.88 - 0.97	0.002
Use of efavirenz (EFV)	0.42	0.22 - 0.78	0.006

a Nucleotide A is the major allele (referent) for rs222020. In a third model, rs222020-G accounts for 3.3% of log_10_ 25(OH)D variance in 93 other subjects (see text).

b Winter is the referent in all tests.

c Favorable effect (association with vitamin D sufficiency) is reflected by a value of >1.0 for the proportional odds ratio (pOR).

### VARIANCE IN log_10_-TRANSFORMED SERUM 25(OH)D CONCENTRATION EXPLAINED BY *GC* GENOTYPES AND OTHER PERTINENT FACTORS

At least six factors independently contributed to the variability in log_10_-transformed serum 25(OH)D concentration (**Table 5**). When sorted by their relative impact (i.e., beta estimate and semi-partial *R*^2^ value), race (AAs vs. other), and season had the greatest effects (adjusted *p* < 0.0001 and *p* < 0.001, respectively), followed by latitude (in three major grids; *p* < 0.001), BMI (*p* = 0.008), use of EFV (*p* = 0.016), and rs222020-G (*p* = 0.023). Collectively, these factors accounted for 38.0% of variance in the log_10_-transformed 25(OH)D concentration at baseline (*p* < 0.0001). Other potential factors, including sex, age, and additional *GC* SNP variants were firmly dismissed (adjusted *p* > 0.25).

**Table 5:** Independent predictors of baseline (week 0) serum 25(OH)D concentration among 192 youth living with HIV-1 infection: alternative analyses after considering environmental factors.

*GC* variant and demographic features	Relative impact^c^ on log_10_ 25(OH)D (multivariable model)		
	Δ (Mean ± SE)	*R*^2^	*p*
rs222020-G^a^	-0.07 ± 0.03	0.018	0.023
Being African–American	-0.15 ± 0.03	0.089	<0.0001
Use of efavirenz (EFV)	-0.07 ± 0.03	0.020	0.016
BMI (per unit change)^b^	-0.01 ± 0.00	0.025	0.008
Latitude^b^	-0.06 ± 0.02	0.045	<0.001
Season^b^	NA	0.073	<0.001
Spring vs. winter	0.12 ± 0.04	NA	0.003
Summer vs. winter	0.19 ± 0.04	NA	<0.0001
Fall vs. winter	0.13 ± 0.04	NA	0.002

a The AA genotype is treated as the referent for rs222020 allele G.

b As defined in **Table 1**.

c For each individual factor, the independent (adjusted) effect size is measured first by the difference (Δ) in serum log_10_ 25(OH)D and then by the R^2^values (all are under-estimated because of partial overlap). For the overall model, R^2^ = 0.380 (p < 0.0001).

Ranking of the six individual (independent) predictors of log_10_-transformed serum 25(OH)D concentration was often complicated by issues with partial overlap. For example, variance explained by the rs222020-G allele varied substantially (from 2.9 to 10.7%) according to the order in which three partially overlapping factors (race, use of EFV, and rs222020-G) were added to the model. In addition, the variance attributable to rs222020-G differed somewhat between AAs (4.5%, adjusted *p* = 0.029) and others (3.3%, adjusted *p* = 0.080) when conditioned on the effect of EFV (**Table 7**). In contrast, after accounting for the effect of rs222020-G, the impact of EFV use on 25(OH)D was only apparent in AAs (adjusted *R*^2^ = 8.8%, *p* = 0.003) and not in others (adjusted *R*^2^ = 0.1%, *p* = 0.812; **Table 7**).

**Table 6:** Three independent interaction terms identified by multivariable analyses.

Interaction terms^a^	Relative impact^d^ on log_10_ 25(OH)D (multivariable model)		
	Δ (mean ± SE)	*R*^2^	*P*
rs222020-G × season	NA	0.055	<0.001
rs222020-G × spring^b^	-0.22 ± 0.05	NA	<0.001
Latitude × season	NA	0.026	0.035
Latitude × fall^c^	-0.11 ± 0.03	NA	<0.001
Latitude × winter^c^	-0.10 ± 0.03	NA	0.002
Use of EFV × race	NA	0.015	0.024

a Non-interactive factors (e.g., BMI) are also included in the model. The AA genotype is treated as the referent for rs222020.

b Seasonality of the rs222020-G effect is restricted to spring.

c Two seasons (fall and winter) capture the main interactive effect of latitude × season.

d For each interaction term, the independent effect size is measured first by the difference (Δ) in serum log_10_ 25(OH)D and then by the R^2^values (all are under-estimated because of partial overlap).

**Table 7:** Examples of racial differences in genetic association with serum 25(OH)D concentration.

*GC* variant and two other factors	Relative impact^c^ on log_10_ 25(OH)D (multivariable model)		
	Δ (Mean ± SE)	*R*^2^	*P*
**Overall cohort (*N* = 192)**			
rs222020-G^a^	-0.09 ± 0.03	0.033	0.004
Being African–American	-0.17 ± 0.03	0.113	2.9 × 10^-7^
Use of efavirenz (EFV)	-0.07 ± 0.03	0.021	0.024
**African–Americans only (*n* = 99)**			
rs222020-G^a^	-0.12 ± 0.05	0.045	0.029
Use of efavirenz (EFV)^b^	-0.14 ± 0.04	0.088	0.003
**Others only (*n* = 93)**			
rs222020-G^a^	-0.07 ± 0.04	0.035	0.071

a Homozygosity with the major allele A serves as the referent for rs222020.

b Not a statistically significant factor in the model for other races (adjusted R^2^ = 0.001, p = 0.812).

c For each factor shown in individual models, the independent effect size is measured first by the difference (Δ) in serum log_10_ 25(OH)D (the reference group is negative for all factors) and then by the R^2^ values.

### INTERACTION TERMS

**FIGURE 3:**
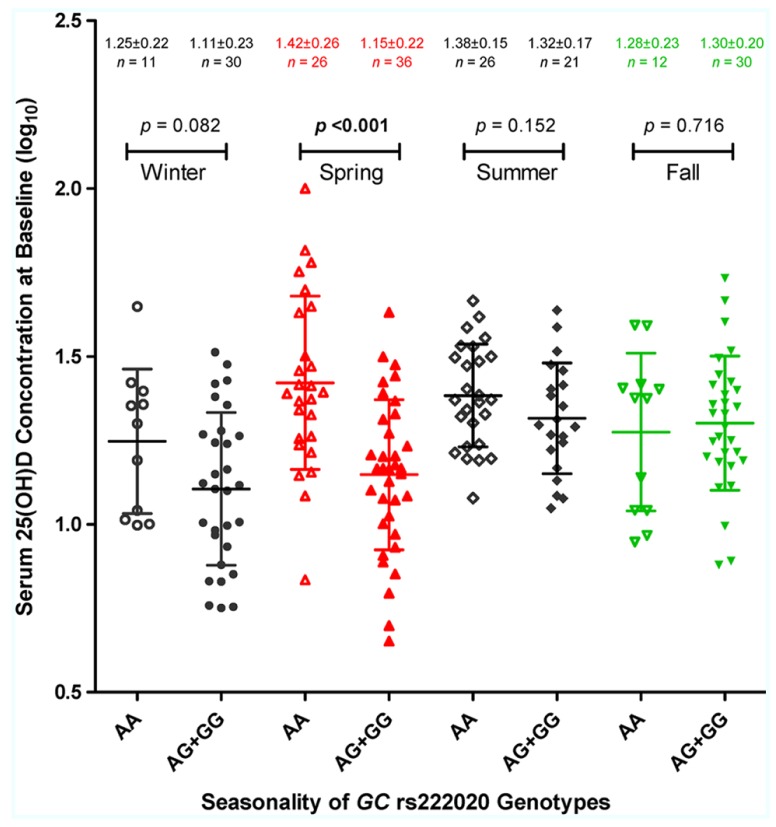
**Season-dependent association of *GC* genotypes with serum 25-hydroxyvitamin D [25(OH)D] concentration.** The log_10_-transformed 25(OH)D (ng/ml) values in 192 HIV-1-infected youth are plotted according to four enrollment seasons and three genotypes defined by the *GC* SNP, rs222020 (major allele A and minor allele G). For each stratum, the horizontal bars connected by a vertical line correspond to the mean ± standard deviation (SD). The nominal *p* values are based on Student’s *t*-test, assuming a dominant effect of allele G (see **Table 6** for full analyses of interactions between rs222020-G and enrollment season).

Multivariable models further revealed several pairwise interactions, i.e., rs222020-G × season (*p* < 0.001), latitude × season (especially fall and winter; *p* < 0.01), and race × EFV use (*p* = 0.024). Seasonality of the rs222020-G effect on 25(OH)D was apparently restricted to spring, as genotype-specific differences were not detected in other seasons (**Figure 3**).

### OBSERVATION OF DIFFERENCES BETWEEN RACIAL GROUPS

At least two racial differences were noted in separate analyses of AAs (*n* = 99) and other races (*n* = 93). First, negative association of rs222020-G with 25(OH)D was restricted to AAs (*R*^2^ = 4.5%, adjusted *p* = 0.029) and not other subjects (3.3%, adjusted *p* = 0.080) when conditioned on the effect of EFV (**Table 7**). Second, the deleterious impact of EFV use on 25(OH)D was seen in AAs (adjusted *R*^2^ = 8.8%, *p* = 0.003) and not in other subjects (adjusted *R*^2^ = 0.1%, *p* = 0.812) after accounting for the contribution of rs222020-G.

## DISCUSSION

Despite a modest sample size, our analyses here reveal five major findings concerning vitamin D metabolism in youth living with HIV-1 infection. First, serum 25(OH)D concentration is relatively stable over a 12-week period regardless of race. Second, at least one *GC* SNP variant, the rs222020-G allele, is independently predictive of suboptimal serum 25(OH)D, especially during the spring season. Third, use of EFV is associated with low serum 25(OH)D in the combined cohort based on univariable models, but the EFV effect is restricted to AAs when the rs222020-G allele is added to multivariable models. Fourth, the exact contribution of genetic and non-genetic factors (latitude, season, BMI, and race) can be obscured by partial overlaps and frequent interactions. Fifth, statistical models are not uniformly applicable to racial groups. Most of these observations are novel and highly relevant to public health.

As a main focus of this study, the *GC* gene^3^ consists of 13 exons and has hundreds of known SNPs, but neither genome-wide association studies nor candidate gene approaches reported in the literature have covered this locus sufficiently enough to allow fine-mapping. To avoid heavy penalty for multiple testing of randomly selected *GC* SNPs, we chose to examine coding and regulatory (promoter) sequences at both ends of several SNPs with relatively consistent associations. For example, the minor allele G (or C in the complementary strand) for rs222020 has been highlighted recently in the context of compression strength index of the femoral neck (Xu et al., 2010), peripheral arthritis in ankylosing spondylitis (Jung et al., 2011), and plasma 25(OH)D concentration (Zhang et al., 2012). By analyzing rs222020 and multiple neighboring SNPs, it was evident that rs222020-G is able to tag several intronic variants within a single haplotype block. However, rs222020-G did not seem to tag other functionally relevant variants. Mechanisms underlying its independent association with suboptimal 25(OH)D concentration remain elusive, and search for further clues may need to consider less obvious pathways (DNA–DNA and DNA–protein interactions) being actively pursued by the ENCODE project (Dunham et al., 2012; Harrow et al., 2012; Rosenbloom et al., 2012; Wang et al., 2013).

Two other prominent *GC* SNPs, rs7041, and rs4588, do cause amino acid substitutions at codon 416 (D/E) and codon 420 (T/K), respectively, in exon 11. Three haplotypes involving these non-synonymous SNPs correspond to different protein isoforms known as GC1F, GC1S, and GC2. Earlier studies have demonstrated the potential importance of rs7041 variants alone (Wood et al., 2011) or in conjunction with rs4588 variants (Fang et al., 2009). Although rs7041-G appeared to be highly favorable in our initial screening (univariable models only), it was subsequently dismissed by multivariable models in which race and other prominent factors were treated as covariates. The distribution of rs7041-G differs between AAs (low) and other races (high; **Table 2**), so definitive analyses may require a third population with intermediate allele frequency. Nonetheless, rs7041-G may serve as a useful biomarker for disparity in serum 25(OH)D concentration, especially since its biological relevance is so obvious. Additional *GC* SNPs of interest, including rs2070741 (Wood et al., 2011) and rs2282679 (Vimaleswaran et al., 2013), are not part of our study design. Judging by their reported effect sizes, it is unlikely that inclusion of these SNPs will alter our main conclusions.

Our finding on *GC* SNP rs222020 here is highly consistent with earlier observations based on two independent Caucasian populations (Bu et al., 2010). The effect size (*R*^2^) reported for rs222020 ranges from 1% to 4% in Caucasians, which is quite similar to what we can demonstrate for YLH (**Table 5**). However, by our assessment, the association of rs222020 genotypes with 25(OH)D concentration is heavily dependent on seasonal fluctuation. Studies that evaluate subjects in the spring season alone can lead to over-estimates, while analyses biased for other seasons can easily miss the genetic effect (**Table 6**; **Figure 3**). Future studies will clearly need to consider the strong impact of season and latitude on vitamin D metabolism.

Recognition of EFV as another factor that influences vitamin D metabolism is also well expected (Childs et al., 2012; Havens et al., 2012a). Of note, the unfavorable impact of EFV was mostly restricted to AAs. Such racial disparities may reflect the sensitivity or vulnerability of AAs to therapeutic complications when they are already prone to having suboptimal 25(OH)D concentration. Fortunately, response to vitamin D supplementation is not compromised by use of EFV (Havens et al., 2012a). Further elucidation of race-specific effects of EFV will need to rely on large cohorts with prospective data.

In summary, our study identified at least six partially overlapping but independent factors that collectively account for 38% of variance in serum 25(OH)D concentration. The complex picture can attest to the need for cautionary interpretation of results from univariable models, as exemplified by analysis of *GC* SNPs rs7401 and rs222020. Fine-mapping of the *GC* locus for causal variants is more difficult than expected because (i) rs222020-G and related *GC* variants are not in clear LD with coding and promoter sequence polymorphisms; (ii) the effect of rs7041-G is clearly confounded by racial background, and (iii) serum 25(OH)D concentration as a quantitative or semi-quantitative trait can fluctuate over time (seasonality). These observations should benefit follow-up studies on *GC* genotypes and vitamin D metabolism, probably beyond the setting of chronic HIV infection and long-term therapeutic complications.

## AUTHOR CONTRIBUTIONS

Charles B. Stephensen, Kathleen Mulligan, Brandy Rutledge, Patricia M. Flynn, Jorge Lujan-Zilbermann, Rohan Hazra, Craig M. Wilson, Peter L. Havens, and Jianming Tang designed the study. Jorge Lujan-Zilbermann helped with patient recruitment and enrollment at one of the sites for Adolescent Medicine Trials Network for HIV/AIDS Interventions. Travis R. Porter, Peter L. Havens, and Jianming Tang procured samples and reagents. Travis R. Porter, Xuelin Li, and Jianming Tang managed and analyzed the data. All authors contributed to the writing and proof reading of this manuscript.

## Conflict of Interest Statement

The authors declare that the research was conducted in the absence of any commercial or financial relationships that could be construed as a potential conflict of interest.

## Footnotes

1 http://www.ncbi.nlm.nih.gov/gene/2638

2 http://clinicaltrials.gov/ct2/show/NCT00490412

3 http://www.ncbi.nlm.nih.gov/gene/2638
